# A New Chromosomal Phylogeny Supports the Repeated Origin of Vectorial Capacity in Malaria Mosquitoes of the *Anopheles gambiae* Complex

**DOI:** 10.1371/journal.ppat.1002960

**Published:** 2012-10-04

**Authors:** Maryam Kamali, Ai Xia, Zhijian Tu, Igor V. Sharakhov

**Affiliations:** 1 Department of Entomology, Virginia Polytechnic Institute and State University, Blacksburg, Virginia, United States of America; 2 Department of Biochemistry, Virginia Polytechnic Institute and State University, Blacksburg, Virginia, United States of America; University of Minnesota, United States of America

## Abstract

Understanding phylogenetic relationships within species complexes of disease vectors is crucial for identifying genomic changes associated with the evolution of epidemiologically important traits. However, the high degree of genetic similarity among sibling species confounds the ability to determine phylogenetic relationships using molecular markers. The goal of this study was to infer the ancestral–descendant relationships among malaria vectors and nonvectors of the *Anopheles gambiae* species complex by analyzing breakpoints of fixed chromosomal inversions in ingroup and several outgroup species. We identified genes at breakpoints of fixed overlapping chromosomal inversions 2Ro and 2Rp of *An. merus* using fluorescence *in situ* hybridization, a whole-genome mate-paired sequencing, and clone sequencing. We also mapped breakpoints of a chromosomal inversion 2La (common to *An. merus*, *An. gambiae*, and *An. arabiensis*) in outgroup species using a bioinformatics approach. We demonstrated that the “standard” 2R+^p^ arrangement and “inverted” 2Ro and 2La arrangements are present in outgroup species *Anopheles stephensi*, *Aedes aegypti*, and *Culex quinquefasciatus*. The data indicate that the ancestral species of the *An. gambiae* complex had the 2Ro, 2R+^p^, and 2La chromosomal arrangements. The “inverted” 2Ro arrangement uniquely characterizes a malaria vector *An. merus* as the basal species in the complex. The rooted chromosomal phylogeny implies that *An. merus* acquired the 2Rp inversion and that its sister species *An. gambiae* acquired the 2R+^o^ inversion from the ancestral species. The karyotype of nonvectors *An. quadriannulatus* A and B was derived from the karyotype of the major malaria vector *An. gambiae*. We conclude that the ability to effectively transmit human malaria had originated repeatedly in the complex. Our findings also suggest that saltwater tolerance originated first in *An. merus* and then independently in *An. melas*. The new chromosomal phylogeny will facilitate identifying the association of evolutionary genomic changes with epidemiologically important phenotypes.

## Author Summary

Malaria causes more than one million deaths every year, mostly among children in Sub-Saharan Africa. *Anopheles* mosquitoes are exclusive vectors of human malaria. Many malaria vectors belong to species complexes, and members within these complexes can vary significantly in their ecological adaptations and ability to transmit the parasite. To better understand evolution of epidemiologically important traits, we studied relationships among nonvector and vector species of the African *Anopheles gambiae* complex. We analyzed gene orders at genomic regions where evolutionary breaks of chromosomal inversions occurred in members of the complex and compared them with gene orders in species outside the complex. This approach allowed us to identify ancient and recent gene orders for three chromosomal inversions. Surprisingly, the more ancestral chromosomal arrangements were found in mosquito species that are vectors of human malaria, while the more derived arrangements were found in both nonvectors and vectors. Our finding strongly suggests that the increased ability to transmit human malaria originated repeatedly during the recent evolution of these African mosquitoes. This knowledge can be used to identify specific genetic changes associated with the human blood choice and ecological adaptations.

## Introduction

Complexes of sibling species are common among arthropod disease vectors [1]–[3]. Members of such complexes are morphologically similar and partially reproductively isolated from each other. The *Anopheles gambiae* complex consists of seven African malaria mosquito sibling species. *Anopheles gambiae* and *An. arabiensis*, the two major vectors of malaria in Africa, are both anthropophilic and can breed in temporal freshwater pools. *Anopheles gambiae* occupies more humid areas, while *An. arabiensis* dominates in arid savannas and steppes. *Anopheles merus* and *An. melas* breed in saltwater, and the habitat of *An. bwambae* is restricted to mineral water breeding sites. These three species are relatively minor malaria vectors mainly due to narrow geographic distributions [4]. *Anopheles quadriannulatus* A and *An. quadriannulatus* B are freshwater breeders and, although to various degrees susceptible to *Plasmodium* infections, are not natural vectors of malaria mainly due to zoophilic behavior [5]–[7]. Inferring the evolutionary history of the *An. gambiae* complex could be crucial for identifying specific genomic changes associated with the human blood choice, breeding site preference, and variations in vector competence. However, the high degree of genetic similarity, caused by the ancestral polymorphism and introgression, complicates the use of molecular markers for the reconstruction of a sibling species phylogeny [8]–[10]. Even the most recent genome-wide transcriptome-based phylogeny reconstruction of multiple *Anophelinae* species could not unambiguously resolve the relationships among *An. gambiae*, *An. arabiensis*, and *An. quadriannulatus* [11].

An alternative approach to inferring the phylogenetic relationships among species is to analyze the distribution of fixed overlapping inversions [4], [7], [12]. This approach is based on the fact that species-specific inversions do not introgress [13] and that inversions are predominantly monophyletic, despite rare occurrences of breakpoint reuse [14]. In addition, chromosomal inversions are more rare events and more consistent characters as compared with nucleotide substitutions [12], [15]. Phylogenies based on inversion data are highly congruent with phylogenies based on DNA sequence data and are shown to be more information rich than are nucleotide data [15]. Members of the *An. gambiae* complex carry 10 fixed inversions that can be used for a phylogeny reconstruction [7]. Five fixed inversions are present on the X chromosome, three inversions are found on the 2R arm, and one is found on each of the 2L and 3L arms (Figure S1) [7]. The only nonvectors in the complex, *Anopheles quadriannulatus* A and B, had been traditionally considered the closest species to the ancestral lineage because they have a large number of hosts, feed on animal blood, tolerate temperate climates, exhibit disjunctive distribution, and possess a “standard” karyotype [4], [7], [16], [17]. More recently, the *An. arabiensis* karyotype had been assumed ancestral because it has the fixed 2La inversion, which was also found in two outgroup species from the Middle Eastern *An. subpictus* complex [18]. Both chromosomal phylogenies assumed the most recent speciation of *An. merus* and an independent origin of the cytologically identical 2La′ inversion in this species [19]. A phylogenetic status of an inversion can be determined more precisely when breakpoints are identified and gene orders across breakpoints are compared between ingroup and multiple outgroup species. The genes found across inversion breakpoints in ingroup and outgroup species are expected to be in their ancestral order [12]. For example, the molecular analysis of the 2La inversion breakpoints and physical mapping of the sequences adjacent to the breakpoints in outgroup species identified the shared 2La inversion in *An. gambiae*, *An. merus*, and *An. arabiensis* and determined the ancestral state of the 2La arrangement [20]–[22].

**Figure 1:**
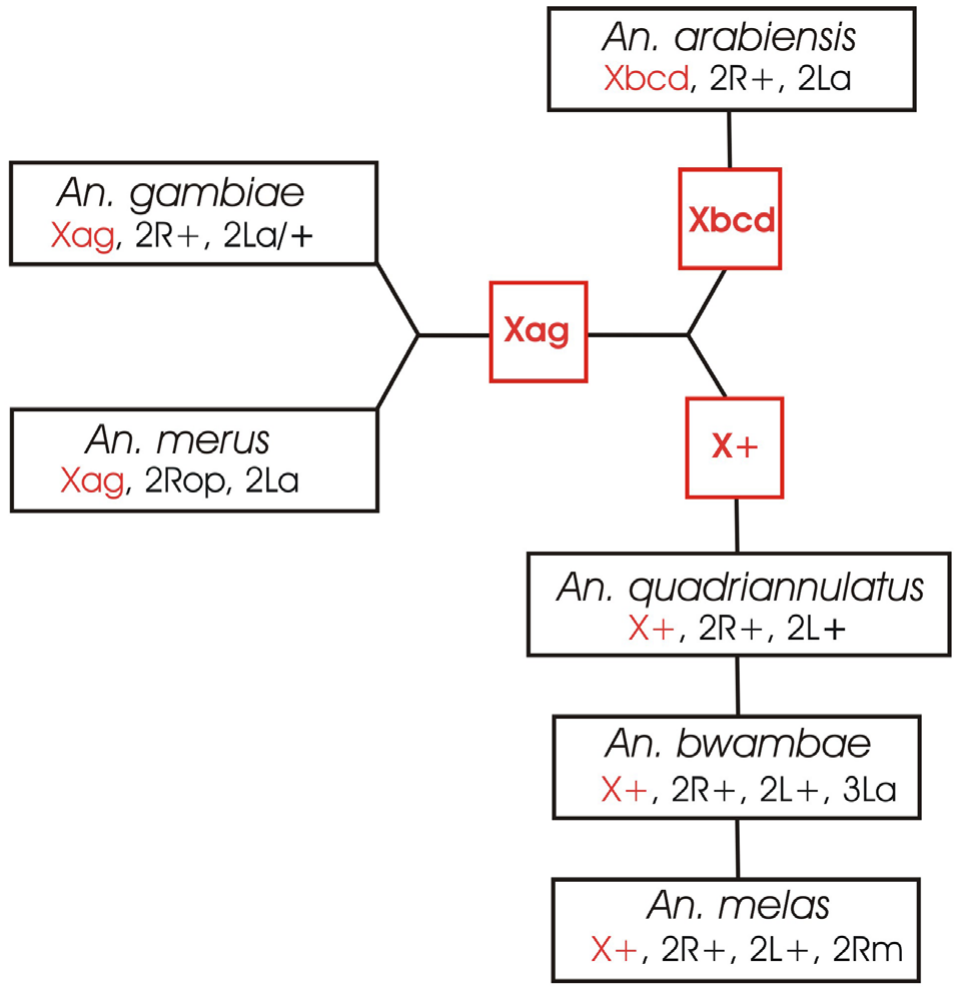
The three species clades identified based on the X chromosome fixed inversions in the *An. gambiae* complex. The X chromosome arrangements are shown in red.

Based on the X chromosome fixed inversions, three species clades can be identified in the complex: (i) *An. bwambae*, *An. melas*, and *An. quadriannulatus* A and B (X+), (ii) *An. arabiensis* (Xbcd), and (iii) *An. merus* and *An. gambiae* (Xag) (Figure 1). The *An. gambiae*–*An. merus* and *An. bwambae*–*An. melas* sister taxa relationships have been supported by independent phylogenetic analyses of nuclear genes and mitochondrial DNA sequences [9], [10], [23]. Each clade has unique fixed inversions that can be used to unambiguously determine its phylogenetic status if compared to gene arrangements in outgroup species: X+, 2Rm, 3La in the *An. bwambae*–*An. melas*–*An. quadriannulatus* clade, Xbcd in *An. arabiensis*, and Xag, 2Ro, 2Rp in the *An. gambiae*–*An. merus* clade. However, to efficiently pursue this research was not possible until recently when genome sequences of several outgroup mosquito species became available, including *An. stephensi* (series *Neocellia*, subgenus *Cellia*, subfamily *Anophelinae*) (this paper), and *Aedes aegypti* and *Culex quinquefasciatus* (both from subfamily *Culicinae*) [24], [25]. In this study, we identified genes at the breakpoints of fixed overlapping inversions 2Ro and 2Rp of *An. merus* and homologous sequences in *An. stephensi*, *Ae. aegypti*, and *C. quinquefasciatus*. We demonstrated that the “inverted” 2Ro and the “standard” 2R+^p^ arrangements are ancestral in the complex. In addition, we found that the “inverted” 2La arrangement is present in evolutionary distant *Culicinae* species and, therefore, is ancestral. The inversion data support the basal position of the *An. gambiae*–*An. merus* clade and the terminal positions of the *An. arabiensis* and *An. melas* lineages. This rooted chromosomal phylogeny could be a means to examine specific genomic changes associated with evolution of traits relevant to vectorial capacity.

## Results/Discussion

To infer the ancestral-descendant relationships among chromosomal arrangements in the *An. gambiae* complex, we determined gene orders at the breakpoints of the *An. merus*-specific fixed overlapping inversions 2Ro and 2Rp in ingroup and several outgroup species, including *An. stephensi*, *Ae. aegypti*, and *C. quinquefasciatus*. In our first approach, we used *An. gambiae* DNA probes, which were identified at breakpoints of “standard” 2R+^o^ and 2R+^p^ arrangements, for the mapping to polytene chromosomes of *An. merus* and *An. stephensi* by fluorescence *in situ* hybridization (FISH). In our second approach, we performed mate-paired sequencing of the *An. merus* genome and mapped the read pairs to the *An. gambiae* AgamP3 genome assembly. The inversion breakpoints of 2Ro and 2Rp in the *An. gambiae–An. merus* clade and their homologous sequences in the outgroup species were obtained and analyzed. This study reconstructed a rooted chromosomal phylogeny and revised evolutionary history of the *An. gambiae* complex.

### Chromosome positions of the 2Ro and 2Rp inversion breakpoints in *An. merus*, *An. gambiae*, and *An. stephensi*

**Figure 2:**
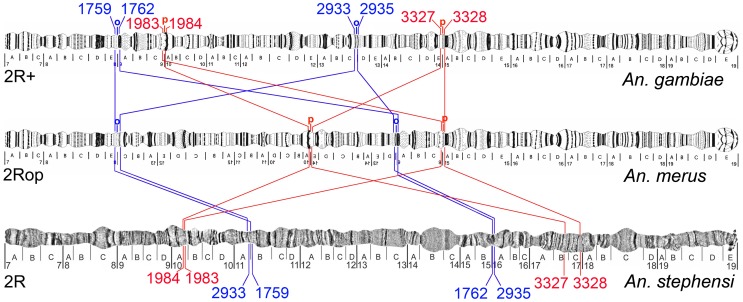
Gene orders in the polytene chromosomes at 2Ro/2R+^o^ and 2Rp/2R+^p^ breakpoints. Genes of ingroup species *An. merus*, *An. gambiae*, and outgroup species *An. stephensi* are shown on polytene chromosomes. Genes AGAP001759, AGAP001762, AGAP002933, and AGAP002933 of 2Ro/2R+^o^ (in blue), and genes AGAP001983, AGAP001984, AGAP003327, and AGAP003328 of 2Rp/2R+^p^ (in red) are indicated by their last four digits.

We mapped multiple *An. gambiae* DNA probes derived from the cytological breakpoints to the chromosomes of *An. merus* by FISH. *Anopheles gambiae* BAC clone 141A14 that spans the proximal 2R+^o^ breakpoint was identified by comparative mapping with *An. merus* in our previous study [21]. FISH of the BAC clone to *An. merus* chromosomes produced two separate signals on 2R indicating an inversion. Reiteration of this procedure with PCR fragments derived from the BAC clone allowed us to localize the breakpoint region within the BAC between genes AGAP002933 and AGAP002935. Further comparative mapping with *An. merus* demonstrated that the distal 2R+^o^ breakpoint in *An. gambiae* is located between genes AGAP001759 and AGAP001762 (Figure S2). We also performed FISH with polytene chromosomes of *An. merus* using multiple probes located near the 2R+^p^ cytological breakpoints of *An. gambiae*. The proximal 2R+^p^ breakpoint was found between genes AGAP003327 and AGAP003328, and the distal 2R+^p^ breakpoint was localized between AGAP001983 and AGAP001984 in *An. gambiae*. These gene pairs were neighboring in the genome of *An. gambiae*, but they were mapped in separate locations in *An. merus* (Figure S3). To determine gene arrangements in an outgroup species, we mapped genes at the 2R+^o^ and 2R+^p^ breakpoints to polytene chromosomes of *An. stephensi* (Figure S4 and Figure S5). The FISH results showed that the “inverted” 2Ro and “standard” 2R+^p^ arrangements are present in the outgroup species *An. stephensi* (Figure 2).

### Structure of the 2Ro and 2R+^o^ inversion breakpoints in *An. merus* and *An. gambiae*

**Figure 3:**
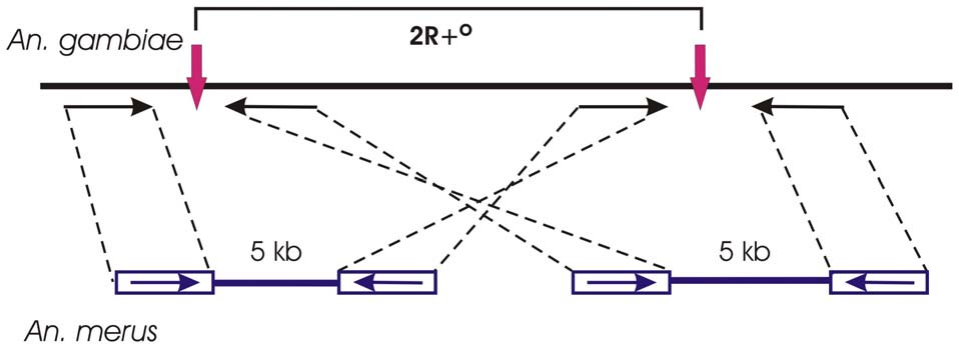
A scheme showing the utility of mate-paired sequencing for identifying inversion breakpoints. The BLASTN search of *An. merus* mate-paired sequencing reads (horizontal arrows) detects the 2R+^o^ inversion breakpoints (vertical arrows) in the *An. gambiae* AgamP3 genome assembly.

We performed mate-paired sequencing of the *An. merus* genome and mapped the read pairs to the *An. gambiae* AgamP3 genome assembly, which has all “standard” arrangements [26], [27]. Mate-paired sequencing is the methodology that enables the generation of libraries with inserts from 2 to 5 kb in size. The 2 kb, 3 kb, and 5 kb DNA fragments were circularized, fragmented, purified, end-repaired, and ligated to Illumina paired-end sequencing adapters. The final libraries consisted of short fragments made up of two DNA segments that were originally separated by several kilobases. These genomic inserts were paired-end sequenced using an Illumina approach. Paired-read sequences that map far apart in the same orientation delineate inversions [28]. We executed a BLASTN search to find read pairs mapped to the putative breakpoint regions in the same orientation on chromosome 2 (Figure 3 and Table S1). Alignment of the read pairs to the genome of *An. gambiae* identified the 2Ro breakpoints at coordinates ∼9.48 Mb and ∼29.84 Mb. We also identified the 2La breakpoints at coordinates ∼20.52 Mb and ∼42.16 Mb, which confirmed a previous study and, thus, validated the approach [20]. However, the BLASTN search did not find the paired-read sequences that map at the opposite 2Rp breakpoints in the same orientation. This approach could not detect breakpoint regions longer than 5 kb. The 2Rp breakpoint regions in *An. merus* likely have larger sizes caused by accumulation of repetitive sequences. We also used the Bowtie program [29] to confirm the genomics positions of the 2Ro breakpoints (Table S2). Both BLASTN and Bowtie results supported the position of the proximal 2Ro breakpoint to the region between genes AGAP001762 and AGAP002935, and they refined the position of the distal 2Ro breakpoint to the region between AGAP001760 and AGAP002933.

**Figure 4:**
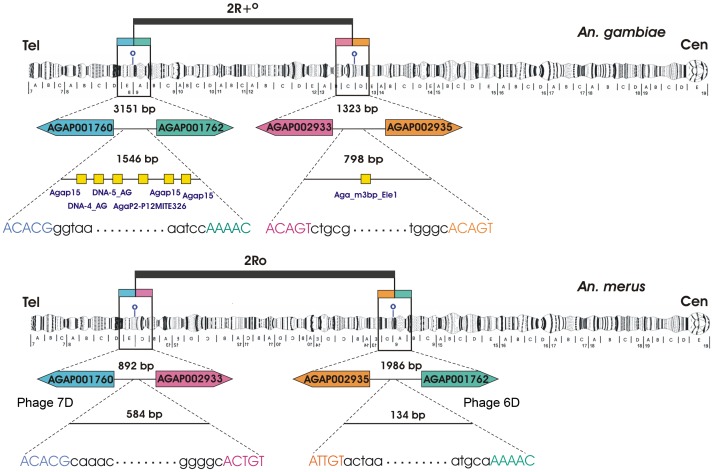
Structure of the 2R+^o^ and 2Ro inversion breakpoint sequences in *An. gambiae* and *An. merus*. Distal and proximal breakpoints are shown on polytene chromosomes and in the *An. gambiae* genome assembly. Breakpoint sequences are shown with small letters, and their sizes are indicated in base pairs. Genes at the breakpoints are shown in their 5′-3′ orientation with boxes of similar colors. Distances between the genes are shown above the intergenic regions. Homologous sequences are represented by identically colored capital letters. Yellow boxes show assemblies of degenerate TEs in *An. gambiae*. The sizes of genes and intergenic regions are not drawn to scale. Cen, centromere. Tel, telomere.

The genes adjacent to the 2Ro breakpoint were used as probes to screen the genomic phage library of *An. merus*. Positive *An. merus* phage clones were confirmed to span inversion breakpoints by FISH to polytene chromosomes of *An. gambiae*, *An. merus*, and *An. stephensi*. For example, hybridization of Phage 6D produced only one signal in the proximal 2Ro breakpoint in *An. merus* but two signals at both 2Ro breakpoints in *An. gambiae* (Figure S6). Phage 6D hybridized to only one locus in *An. stephensi*, confirming the 2Ro arrangement in this species. Confirmed phage clones were sequenced, and the exact breakpoint regions were identified by aligning the *An. merus* sequences and *An. gambiae* AgamP3, AgamM1, and AgamS1 genome assemblies available at VectorBase [26], [30], [31]. Thus, distal and proximal breakpoints were identified on polytene chromosome map [7] and in the genome assembly of *An. gambiae* (Figure 4). In the AgamP3 assembly, the distal and proximal breakpoint regions span coordinates 9,485,167–9,486,712, and 29,838,366–29,839,163, respectively. The 2Ro breakpoint regions were 2.6 and 5.9 times smaller in *An. merus* as compared with the 2R+^o^ breakpoint regions in *An. gambiae* due to accumulation of transposable elements (TEs) in the latter species. The presence of TEs is a common signature of inversion breakpoints, as TEs usually mark breakpoints of derived arrangements [20], [32]. Five various DNA transposons were found at the distal 2R+^o^ breakpoint, and one novel miniature inverted-repeat TE (MITE), Aga_m3bp_Ele1, was identified at the proximal 2R+^o^ breakpoint in *An. gambiae* (Figure 4). Smaller sizes of the breakpoint regions and the lack of TEs at the breakpoints of *An. merus* strongly suggest the ancestral state of the 2Ro arrangement.

### Gene orders at the 2Ro, 2Rp, and 2La inversion breakpoints in outgroup species

**Figure 5:**
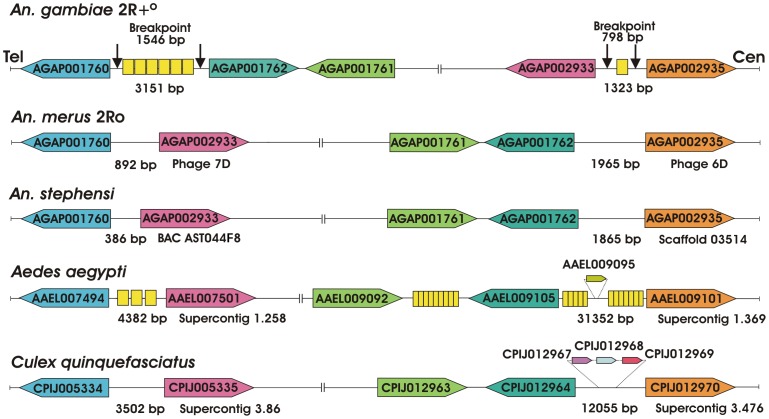
Gene orders in assembled sequences of the 2R+^o^ and 2Ro breakpoints. Genes of *An. gambiae* and *An. merus* as well as three outgroup species *An. stephensi*, *Ae. aegypti*, and *C. quinquefasciatus* are shown. Breakpoint regions in *An. gambiae* are represented by vertical black arrows with their sizes in base pairs. Homologous genes are show in their 5′-3′ orientation with boxes of similar colors. Distances between genes are shown in base pairs and are not depicted proportionally. The correct orientation of genes with respect to the centromere (Cen) and telomere (Tel) is shown only for *An. gambiae*. Additional genes at the breakpoints of *Ae. aegypti* and *C. quinquefasciatus* are shown in a smaller scale. Yellow boxes show assemblies of degenerate TEs. In the *An. gambiae* breakpoints, TEs are shown in the following order from left to right: AgaP15, DNA-4_AG, DNA-5_AG, AgaP2-P12MITE326, AgaP15, AgaP15 (distal breakpoint), and Aga_m3bp_Ele1 (proximal breakpoint). The sizes of genes and intergenic regions are not drawn to scale.

We determined gene orders at the breakpoints of the *An. merus*-specific fixed overlapping inversions 2Ro and 2Rp in several outgroup species, including *An. stephensi*, *Ae. aegypti*, and *C. quinquefasciatus*. The genes adjacent to the 2Ro and 2Rp breakpoint were used as probes to screen the genomic BAC library of the outgroup species *An. stephensi*. Sequences homologous to genes from the distal 2Ro breakpoint were found in the BAC clone AST044F8 of *An. stephensi*. In addition, we performed sequencing of the *An. stephensi* genome using 454 and Illumina platforms. Sequences homologous to genes from the proximal 2Ro breakpoint were identified in scaffold 03514 of the *An. stephensi* genome. We also detected homologous sequences in the genome assemblies of *Ae. aegypti* and *C. quinquefasciatus* available at VectorBase [27]. The analysis demonstrated that all studied outgroup species had the gene arrangement identical to that of *An. merus* confirming the ancestral state of the 2Ro inversion (Figure 5). The *An. stephensi* sequences, which correspond to the 2Ro breakpoints, had sizes more similar to those in *An. merus* than in *An. gambiae*, and they did not display any TEs or repetitive elements, further supporting the 2Ro ancestral state. However, we found TEs in sequences corresponding to one of the 2Ro breakpoints in *Ae. aegypti*. Incidentally, the areas between the homologous breakpoint-flanking genes were 12,055 bp in *Culex* and 31,352 bp in *Aedes*, and this probably reflects the repeat-rich nature of the *Culicinae* genomes. The demonstrated conservation of gene orders between *Anophelinae* and *Culicinae* species is remarkable given the ∼145–200 million years of divergence time between these two lineages [33].

**Figure 6:**
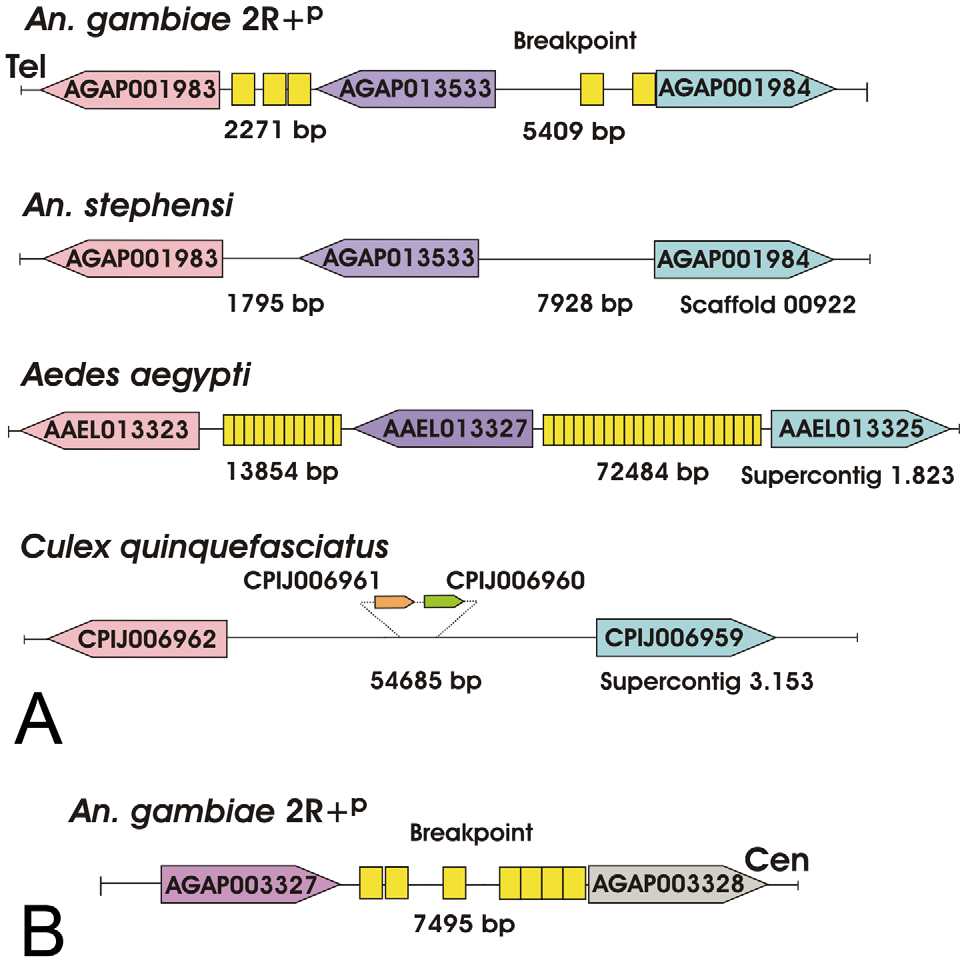
Gene order in assembled sequences of the 2R+^p^ breakpoints. Genes of *An. gambiae* as well as three outgroup species *An. stephensi*, *Ae. aegypti*, and *C. quinquefasciatus* are shown. (**A**) The distal 2R+^p^ breakpoint region. Distances between genes are indicated in base pairs, and they are not depicted proportionally. Homologous genes are shown in their 5′-3′ orientation with boxes of similar colors. Additional genes at the breakpoint of *C. quinquefasciatus* are shown in a smaller scale. Yellow boxes show assemblies of degenerate TEs. In *An. gambiae*, TEs are shown in the following order from left to right: SINEX-1_AG, P4_AG, SINEX-1_AG, RTE-1_AG, and SINEX-1_AG. (**B**) The proximal 2R+^p^ breakpoint region. The *An. stephensi*, *Ae. aegypti*, and *C. quinquefasciatus* genes homologous to genes from the proximal 2R+^p^ breakpoint of *An. gambiae* are found in different scaffolds and supercontigs and, therefore, are not shown. In *An. gambiae*, TEs are shown in the following order from left to right: AARA8_AG, CR1-8_AG, Copia-6_AG-LTR, Clu-47_AG, Clu-47_AG, SINEX-1_AG, and Clu-47_AG. The sizes of genes and intergenic regions are not drawn to scale. The correct orientation of genes with respect to the centromere (Cen) and telomere (Tel) is shown only for *An. gambiae*.

Approximate genomic positions of the 2R+^p^ breakpoints were determined between AGAP001983 and AGAP001984 and between AGAP003327 and AGAP003328 by physical mapping of *An. merus* chromosomes (Figure 2). Using these genes as probes, we obtained a positive Phage 3B of *An. merus* that was mapped to the proximal 2Rp breakpoint in *An. merus* (Figure S6). Sequencing and molecular analyses of Phage 3B revealed the presence of AGAP001983 and AGAP013533 in this clone indicating that the actual distal breakpoint is located between AGAP013533 and AGAP001984 in *An. gambiae*. However, the available Phage 3B sequence ended at gene AGAP013533 and, thus, did not encompass the actual breakpoint sequence in *An. merus*. We performed the comparative analysis of gene orders at the 2Rp breakpoints in three outgroup species, *An. stephensi*, *C. quinquefasciatus*, and *Ae. aegypti*. The results demonstrated the common organization of the distal 2R+^p^ breakpoint in *An. gambiae* and outgroup species, indicating that this arrangement is ancestral (Figure 6). Interestingly, a gene similar to AGAP013533 was absent, but genes similar to AGAP001983 and AGAP001984 were present in supercontig 3.153 of *C. quinquefasciatus*. Genes similar to AGAP003327 and AGAP003328 were found in different scaffolds and supercontigs of the outgroup species. This pattern was expected because AGAP003327 and AGAP003328 were mapped to neighboring but different subdivisions on the *An. stephensi* chromosome map (Figure 2). Therefore, it is possible that an additional inversion separated these two genes in the *An. stephensi* lineage. The highly fragmented nature of the *C. quinquefasciatus* and *Ae. aegypti* genome assemblies could also explain the observed pattern. No TEs were found in the breakpoint regions of *An. stephensi* and *C. quinquefasciatus*. However, multiple TEs were found in the intergenic regions of *An. gambiae* and *Ae. aegypti* (Figure 6).

Using sequencing and cytogenetic approaches, the common 2La arrangement was previously found in *An. gambiae*, *An. merus*, and *An. arabiensis* [4], [20], as well as in several outgroup species, including *An. subpictus* [18], *An. nili*, and *An. stephensi* [22]. Here, we used sequences available for breakpoints of the 2La inversion [20] to execute BLAST searches against genomes of more distantly related outgroup species *C. quinquefasciatus* and *Ae. aegypti*. BLAST results of genes adjacent to the 2La proximal breakpoint, AGAP007068 and AGAP005778, identified orthologs CPIJ004936 and CPIJ004938 in the *Culex* genome as well as orthologs AAEL001778 and AAEL001757 in the *Aedes* genome. These genes were found within supercontig 3.77 in *C. quinquefasciatus* and within supercontig 1.42 in *Ae. aegypti*. Similarly, BLAST results of genes neighboring with the 2La proximal breakpoint, AGAP007069 and AGAP005780, identified homologous genes CPIJ005693 and CPIJ005692 in the *Culex* genome (supercontig 3.99) as well as AAEL011139 and AAEL011140 in the *Aedes* genome (supercontig 1.543). The obtained data confirmed the identical gene arrangement in distant outgroup species and the ancestry of the 2La inversion.

### Chromosomal phylogeny of the *An. gambiae* complex

**Figure 7:**
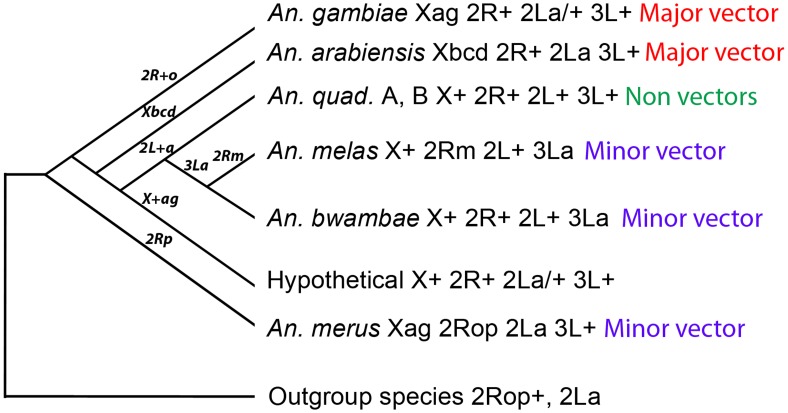
A rooted chromosomal phylogeny of the *An. gambiae* complex. The phylogeny is based on the ancestry of the 2Ro, 2R+^p^, and 2La arrangements found in outgroup species. The vector status for each species is indicated. Inversion fixation events are shown above the branches.

Physical chromosome mapping and bioinformatic analyses identified the 2Ro and 2R+^p^ arrangements in several outgroup species indicating that these arrangements are ancestral (Figure 5 and Figure 6). Because these two inversions overlap, only certain evolutionary trajectories and inversion combinations are possible (Figure 2). Specifically, the 2Rop–2Ro+^p^–2R+^op^ order of inversion events is possible, while the 2Rop–2R+^o^p–2R+^op^ evolutionary sequence is not possible, regardless of the direction. Identification of 2Ro and 2R+^p^ as the ancestral arrangements agrees well with this argument. We have also examined three different scenarios in reconstructing chromosomal phylogeny based on the established ancestry of 2Ro, 2R+^p^, and 2La and on the alternative hypothetical ancestries of X chromosomal arrangements (X+, Xag, or Xbcd) using the Multiple Genome Rearrangements (MGR) program [34]. Three different X chromosome arrangements (X+, Xag, and Xbcd) in an outgroup species were examined (Figure S7). The MGR program calculated the phylogenetic distances among species related to the ancestry of the X chromosome arrangement. Three hypothetical trees were obtained and used for interpretation of phylogenetic relationship and inversion reuse in the complex. Of the three scenarios, only the phylogeny based on the ancestry of 2Ro, 2R+^p^, 2La, and Xag had all inversions originating only once in the evolution of the *An. gambiae* complex. The other scenarios (with X+ and Xbcd being ancestral) had multiple origins of one of the inversions implying that they are less parsimonious (Figure S7). Because Xag uniquely characterize the *An. gambiae*–*An. merus* clade, these two species have the least chromosomal differences from the ancestral species of the complex as compared with other members (Figure 7). The ancestry of Xag can be tested by mapping of the X chromosome genome sequences from several species of the *An. gambiae* complex, which soon will be available [10]. Importantly, the new phylogeny is in complete agreement with the previous discoveries of 2La being the ancestral arrangement [18], [20]. Moreover, this is the first phylogeny based on knowledge about the status of a species-specific inversion (2Ro of *An. merus*). Therefore, the future data on the ancestry of the X chromosome arrangement are expected to support the new phylogeny.

### Hypothetical evolutionary history of the *An. gambiae* complex

**Figure 8:**
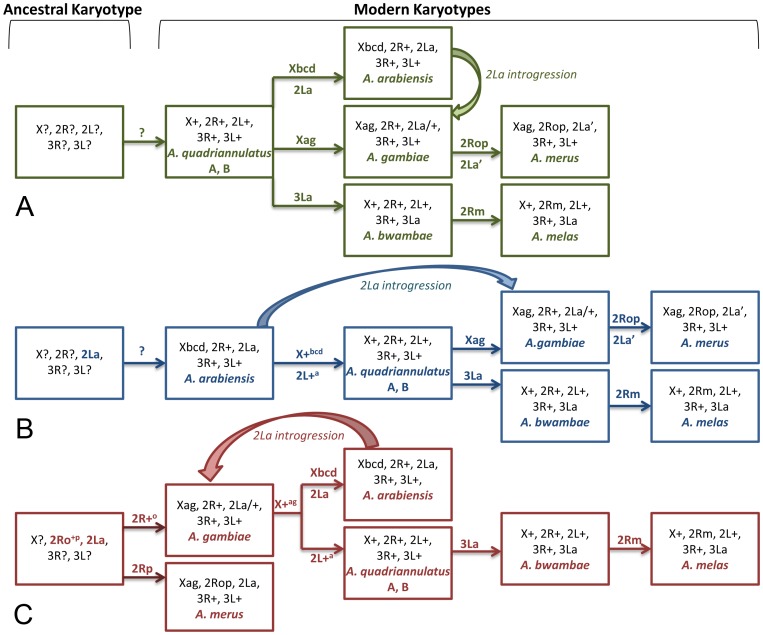
Alternative scenarios of karyotypic evolution in the *An. gambiae* complex. (**A**) A chromosomal phylogeny based on the ancestral state of the “standard” karyotype of *An. quadriannulatus* [4], [7]. (**B**) A karyotypic evolution based on the ancestral position of the *An. arabiensis* karyotype inferred from the finding of the fixed 2La inversion in outgroup species [18]. Scenarios A and B assume an independent origin of the 2La′ inversion in *An. merus*. (**C**) A chromosomal phylogeny based on the established ancestry of the shared inversion 2La [20] and arrangements 2Ro and 2R+^p^ (this study). The introgression of 2La from *An. arabienstis* to *An. gambiae* is shown in all three scenarios. Inversion fixation events are shown above and below arrows.

Speciation in the *An. gambiae* complex has been accompanied by fixation of chromosomal inversions, except for speciation within the *An. quadriannulatus* lineage [7], [35]. Therefore, the chromosomal phylogeny likely reflects the species' evolutionary history. For a long time, the *An. quadriannulatus* lineage had been traditionally considered ancestral [4], [7], [16], [17] (Figure 8A). This evolutionary history was reconstructed from an unrooted phylogeny without any knowledge about chromosomal arrangements in outgroup species. Later, the *An. arabiensis* lineage had been assumed basal because it has the fixed ancestral 2La inversion and based on knowledge about biogeography and ecology of *An. arabiensis* [18] (Figure 8B). In these two scenarios, saltwater species *An. merus* and *An. melas* had been assumed the most recently originated members in the complex. However, the ancestry and the unique origin of the 2La inversion [20] imply that *An. arabiensis*, *An. gambiae*, or *An. merus* could be the closest to the ancestral species. The new chromosomal phylogeny led us to the substantial revision of the evolutionary history of the *An. gambiae* complex (Figure 8C). Accordingly, the ancestral species with 2Ro, 2R+^p^, and 2La arrangements might have arisen in East Africa where *An. merus* and *An. gambiae* are present in sympatry. The ancestral species may have been polymorphic for the 2Rp and 2R+^o^ inversions and one lineage or population gave rise to *An. merus* with the 2Rp inversion while the other gave rise to the sister species *An. gambiae* containing the 2R+^o^ inversion. Otherwise one would have to postulate that *An. gambiae* and *An. merus* arose from independent ancestors. At some point in evolutionary history, *An. gambiae* acquired polymorphic 2La/+ inversion and entered forested regions in central Africa. Later, *An. gambiae* acquired multiple polymorphic inversions on 2R, which allowed this species to spread to the arid areas of West Africa [4]. A hypothetical karyotype might have originated from the *An. gambiae* chromosomal arrangements by acquiring X+^ag^ inversions. This karyotype in turn gave rise to the *An. arabiensis* chromosomes by generating the Xbcd inversions and fixing 2La and to the *An. quadriannulatus* karyotype by fixing the 2L+^a^ arrangement. The 3La inversion in *An. bwambae* originated from the *An. quadriannulatus* karyotype, followed by the origin of the 2Rm inversion in *An. melas*.

The two major malaria vectors *An. arabiensis* and *An. gambiae* are sympatric species in most of their distribution range, allowing for introgressive hybridization between them. Available data support the hypothesis of introgression of the 2La arrangement from *An. arabiensis* into *An. gambiae* [9], [36], [37]. According to the new chromosomal phylogeny, introgression of 2La has been happening from the more derived karyotype of *An. arabiensis* to the more ancestral karyotype of *An. gambiae*. Therefore, the 2La arrangement in isolated *An. gambiae* populations must retain alleles that are more distantly related to alleles of the 2La arrangement in *An. arabiensis*. This hypothesis can be tested by the genomic analysis of *An. gambiae* island populations that do not have a history of hybridization with *An. arabiensis*. Because the 2La inversion in *An. gambiae* mainland populations has been associated with a tolerance to aridity and slightly reduced susceptibility to *Plasmodium falciparum* [4], [38], [39], the expected differences between the “original” and “introgressed” 2La arrangements could impact our understanding of a role of the inversion polymorphism in mosquito adaptation and malaria transmission.

### Repeated origin of vectorial capacity and ecological adaptations

The results of this study indicate that *An. merus* is closely related to an ancestral species from which the *An. gambiae* complex arose. *Anopheles merus* is a minor vector of human malaria in African mainland. A role of *An. merus* in malaria transmission in Madagascar has also been documented [40]. Based on the unique origin of fixed inversions and X-linked sequences, *An. merus* and *An. gambiae* are considered sister taxa [9], [10]. Therefore, according to the new chromosomal phylogeny, these two species possess the most “primitive” karyotypes in the complex. Our data suggest that the major malaria vector in Africa *An. gambiae* could be more closely related to the ancestral species than was previously assumed. Unexpectedly, we found that the karyotype of nonvectors *An. quadriannulatus* A and B was derived from the karyotype of *An. gambiae* (Figure 7 and Figure 8). *Anopheles quadriannulatus* is not involved in malaria transmission in nature due to its strong preference for feeding on animals [7]. *Anopheles melas* has the most recently formed karyotype and is a malaria vector in West Africa [41], [42].

The new chromosomal strongly suggests that vectorial capacity evolved repeatedly in the *An. gambiae* complex. Increased anthropophily could not have evolved in *An. gambiae* and *An. arabiensis* before humans originated and evolved to high enough densities. Therefore, the ability to effectively transmit human malaria must be a relatively recent trait in the complex. If *An. quadriannulatus* were the ancestral species, as it was assumed earlier [4], [7], then vectorial capacity could have originated only once when all other members split from the *An. quadriannulatus* lineage (Figure 8A). However, if the *An. gambiae*–*An. merus* clade is ancestral, as we demonstrated here, then vectorial capacity must have arisen independently in different lineages after the species were diversified. The available data cannot clearly delineate between the loss of vectorial capacity in *An. quadriannultus* and its subsequent reappearance in *An. bwambae* and *An. melas* with a possible alternative that vectorial capacity in present day *An. quadriannulatus* was only lost after *An. bwambae* and *An. melas* split from the *An. quadriannulatus* lineage. Depending on when the phenotypic change occurred (before or after *An. bwambae*/*An. melas* split from the *An. quadriannulatus* lineage) different scenarios are possible. However, even if a zoophilic behavior was acquired by *An. quadriannulatus* after the split from *An. bwambae* and *An. melas*, one still has to assume repeated origin of vectorial capacity. In this case, it originated independently in *An. gambiae*, *An. merus*, *An. arabiensis*, and the lineage that led to *An. quadriannulatus*/*An. bwambae*/*An. melas*. This alteration of the phylogeny of the *An. gambiae* species complex will likely have direct impact on studies aimed at understanding the genetic basis of traits important to vectorial capacity.

The chromosomal phylogeny also supports the idea of multiple origins of similar ecological adaptations in the complex. An early cytogenetic and ecological study postulated the repeated evolution of saltwater tolerance in the complex [4]. *Anopheles melas* and *An. merus* breed in saltwater pools in western and eastern Africa, respectively. Our finding revealed that the physiological adaptation to breeding in saltwater originated first in *An. merus* and then independently in *An. melas*.

### Conclusion

Because of the high degrees of genetic similarities among sibling species, attempts to use molecular markers to reconstruct phylogenetic trees often fail [10]. Our study provides the methodology for rooting chromosomal phylogenies of sibling species complexes, which are common among disease vectors, including blackflies, sandflies, and mosquitoes [1]–[3]. The robustness of this methodology is supported by the agreement between the two alternative approaches to breakpoint mapping (cytogenetics and sequencing) and by the consensus among the three inversions in the phylogenic analysis (2Ro, 2Rp, and 2La). As we demonstrated, inversion breakpoints can be physically mapped on polytene chromosomes by FISH and identified within genomes by mate-pair and clone sequencing. Importantly, the increasing availability of sequenced and assembled genomes provides an opportunity for identification of gene orders in multiple outgroup species for rooting chromosomal phylogenies.

The high genetic similarity among the species of the *An. gambiae* complex suggests their recent evolution [10], [18]. The identified chromosomal relationships among the species demonstrate rapid gains and losses of traits related to vectorial capacity and ecological adaptations. This study reinforces the previous observations that vectors often do not cluster phylogenetically with nonvectors [1], [10]. The genome sequences for several members of the *An. gambiae* complex are soon to be released [10], and the new chromosomal phylogeny will provide the basis for proposing hypotheses about the evolution of epidemiologically important phenotypes. An intriguing question is whether or not evolution of independently originated traits, such as anthropophily and salt tolerance, is determined by changes of the same genomic loci in different species. In addition, the revised phylogeny will affect the interpretation of results from population genetics studies such as shared genetic variation and the detection of signatures of selection. Specifically, variations shared with *An. merus* but not with *An. quadriannulatus* would be interpreted now as ancestral. Knowledge about how evolutionary changes related to ecological and behavioral adaptation and how susceptibility to a pathogen in arthropod vectors had happened in the past may inform us about the likelihood that similar changes will occur in the future.

## Materials and Methods

### Mosquito strains and chromosome preparation

The OPHASNI strain of *An. merus*, the Indian wild-type laboratory strain of *An. stephensi*, and the SUA2La strain of *An. gambiae* were used for chromosome preparation. To obtain the polytene chromosomes, ovaries were dissected from half-gravid females and kept in Carnoy's fixative solution (3 ethanol: 1 glacial acetic acid) in room temperature overnight. Follicles of ovaries were separated in 50% propionic acid and were squashed under a cover slip. Slides with good chromosomal preparations were dipped in liquid nitrogen. Then cover slips were removed, and slides were dehydrated in a series of 50%, 70%, 90%, and 100% ethanol.

### FISH

Multiple *An. gambiae* DNA probes derived from the cytological breakpoints of *An. gambiae* were physically mapped to the chromosomes of *An. merus* and *An. stephensi*. DNA probes obtained from PCR products were labeled by the Random Primers DNA Labeling System (Invitrogen Corporation, Carlsband, CA), and phage clones were labeled by the Nick Translation Kit (Amersham, Bioscience, Little Chalfont Buckinghamshire, UK). DNA probes were hybridized to chromosome slides overnight at 39°C. Then chromosomes were washed with 1× SSC at 39°C and room temperature. Chromosomes were stained with 1 mM YOYO-1 iodide (491/509) solution in DMSO (Invitrogen Corporation, Carlsbad, CA, USA) and were mounted in DABCO (Invitrogen Corporation, Carlsbad, CA, USA). Images were taken by a laser scanning microscope and by the fluorescent microscope. Location of the signals was determined by using a standard photomap of *An. stephensi* [43] and *An. gambiae* [44].

### Genome sequencing

Mate-paired whole genome sequencing was done on genomic DNA isolated from five adult males and females of *An. merus*. Genomic DNA of *An. merus* was isolated using the Blood and Cell Culture DNA Mini Kit (Qiagen Science, Germantown, MD, USA). Three libraries of 2 kb, 3 kb, and 5 kb were obtained. These libraries were used for 36 bp paired-end sequencing utilizing the Illumina Genome Analyzer IIx at Ambry Genetics Corporation (Aliso Viejo, CA, USA). The 16× coverage genome assembly for *An. stephensi* was obtained by sequencing genomic DNA isolated from Indian wild-type laboratory strain. The sequencing was done using Illumina and 454 platforms at the Core Laboratory Facility of the Virginia Bioinformatics Institute, Virginia Tech.

### Phage and BAC library screening

Screening the *An. merus* Lambda DASH II phage library with genes adjacent to standard 2R+^o^ and 2R+^p^ was performed. To prepare probes for screening phage and BAC libraries, genomic DNA of *An. gambiae* was prepared using the Qiagen DNeasy Blood and Tissue Kit (Qiagen Science, Germantown, MD, USA). Primers were designed for genes adjacent to breakpoints using the Primer3 program [45]. PCR conditions were the following: 95°C for 4 min; 35 cycles of 94°C for 30 s, 55°C for 30 s, and 72°C for 30 s; and 72°C for 5 min. All PCR products were purified from the agarose gel using GENECLEAN III kit (MP Biomedicals, Solon, OH, USA). DNA probes were labeled based on random primer reaction with DIG-11-dUTP from DIG DNA Labeling Kit (Roche, Indianapolis, IN, USA). *Anopheles merus* Lambda DASH II phage library and *An. stephensi* BAC library (Amplicon Express, Pullman, WA, USA) were screened. Library screening was performed using the following kits and reagents (Roche Applied Science, Indianapolis, IN) according to protocols supplied by the manufacturer: Nylon Membranes for Colony and Plaque Hybridization, DIG easy Hyb, DIG Wash and Block Buffer Set, Anti-Dioxigenin-AP, and CDP Star ready to use. Positive phages were isolated with Qiagen Lambda midi Kit (Qiagen Science, Germantown, MD, USA), and positive BAC clones were isolated using the Qiagen Large Construct Kit (Qiagen Science, Germantown, MD, USA).

### Clone sequencing

Primers 1760RCL (5′AGCAACAGGGACGATTTGTT3′) and 2933RCL (5′CTCGCTTTGGTTTGTGCTTT3′) were designed based on AGAP001760 and AGAP002933 sequences, and they were used to obtain the distal 2Ro breakpoint from Phage 7D DNA. The PCR conditions with Platinum *PfX* DNA polymerase (Invitrogen, Carlsbad, CA, USA) were: 94°C for 2 min; 35 cycles of 94°C for 15 s, 55°C for 30 s, and 68°C for 2 min; and 68°C for 10 min. Sanger sequencing of Phage 7D was performed using an ABI machine at the Core Laboratory Facility of the Virginia Bioinformatics Institute, Virginia Tech. Other positive phage and BAC clones were completely sequenced by the paired-end approach using an Illumina platform. Libraries of phages and BAC clones were made using Multiplex Sample Preparation Oligonucleotide Kit and Paired End DNA Sample Prep Kit (Illumina, Inc., San Diego, CA). Paired-end sequencing was performed on the Illumina Genome Analyzer IIx using 36 bp paired-end processing at Ambry Genetics Corporation (Aliso Viejo, CA, USA).

### Bioinformatics analysis

Phage clone of *An. merus*, BAC clone of *An. stephensi*, and genome sequences *of An. merus*, *An. stephensi*, *An. gambiae*, *C. quinquefasciatus*, and *Ae. aegypti* were analyzed with BLASTN, TBLASTX, and BLAST2 using the laboratory server and the Geneious 5.1.5 software (www.geneious.com), a bioinformatics desktop software package produced by Biomatters Ltd. (www.biomatters.com). Identification of the accurate breakpoint was performed by aligning the *An. merus* sequences and *An. gambiae* AgamP3, AgamM1, and AgamS1 genome assemblies available at VectorBase [27]. The DNA transposons and retroelements were analyzed by using the RepeatMasker program [46] and by comparing to Repbase [47] and TEfam (http://tefam.biochem.vt.edu/tefam/) databases. To characterize novel TEs in the breakpoint, each candidate sequence was used as a query to identify repetitive copies in the genome using BLASTN searches. These copies, plus 1000 bp flanking sequences, were aligned using CLUSTAL 2.1 to define the 5′ and 3′ boundaries. Using this approach, a novel MITE was discovered in the *An. gambiae* breakpoint. According to the TEfam naming convention, this MITE was named Aga_m3bp_Ele1 because it was associated with a 3 bp target site duplication.

### Accession numbers

All sequence data have been deposited at the National Center for Biotechnology Information short read archive (www.ncbi.nlm.nih.gov/Traces/sra/sra.cgi) as study no. SRP009814 of submission no. SRA047623 and to the GenBank database (http://www.ncbi.nlm.nih.gov/Genbank/) as accession nos.: JQ042681–JQ042688.

## Supporting Information

Figure S1 **The 10 fixed paracentric inversions in sibling species of the** ***An. gambiae*** **complex.** The positions of breakpoints are shown in blue with small letters above the chromosomes.
(TIF)

Figure S2 **Physical mapping of genes at the 2Ro inversion breakpoints on polytene chromosomes of** ***An. merus*** **.** **A**) FISH of AGAP001759 (blue signal) and AGAP001762 (red signal) to subdivisions 8E and 9A, which are located at the distal and proximal breakpoints, respectively. **B**) Localization of AGAP002933 (red signal) in the distal breakpoint (13C) and AGAP002935 (blue signal) in the proximal breakpoint (13D). Arrows point at the hybridization signals. Arrowheads show additional signals from AGAP001762. Chromosomes are counterstained with the fluorophore YOYO-1.
(TIF)

Figure S3 **Physical mapping of genes at the 2Rp inversion breakpoints on polytene chromosomes of** ***An. merus*** **.** **A**) FISH of AGAP001983 (red signal) and AGAP001984 (blue signal) to subdivisions 9C and 10A, which are located at the proximal and distal breakpoints, respectively. **B**) Localization of AGAP001983 (blue signal) and AGAP003328 (red signal) in the neighboring subdivisions 9C and 15A of the proximal breakpoint. **C**) FISH of AGAP003327 (red signal) with the distal breakpoint (10A) and of AGAP001982, the neighboring gene of AGAP001983, (blue signal) with the proximal breakpoint (9C). **D**) Mapping of AGAP001984 (blue signal) to the distal breakpoint (14E) and of AGAP003328 (red signal) to the proximal breakpoint (15A). Arrows point at the hybridization signals. Arrowhead shows an additional signal from AGAP003327.
(TIF)

Figure S4 **Physical mapping of genes from the 2Ro inversion breakpoints on polytene chromosomes of** ***An. stephensi*** **.** **A**) FISH of AGAP001759 (blue signal) to subdivision 11AB. **B**) Localization of AGAP001762 (blue signal) in subdivision 15B-16A. **C**) FISH of AGAP002933 (red signal) with subdivision 11AB and of AGAP002935 (blue signal) in subdivision 15B-16A. **D**) Colocalization of probes derived from transcripts AGAP002933-RA (red signal) and AGAP002933-RB (blue signal) in subdivision 11AB. Arrows point at the hybridization signals.
(TIF)

Figure S5 **Physical mapping of genes from the 2Rp inversion breakpoints on polytene chromosomes of** ***An. stephensi*** **.** **A**) FISH of AGAP001983 (blue signal) and AGAP003328 (red signal) to subdivisions 10A and 17C, respectively. **B**) Localization of AGAP003327 (blue signal) in subdivision 17B. **C**) FISH of AGAP001984 (blue signal) to subdivision 10A and of AGAP003326, the neighboring gene of AGAP003327, (red signal) to subdivision 17B. **D**) Mapping of AGAP001981, a gene located in the vicinity of AGAP001983, (red signal) in subdivision 10A and of AGAP003322, a gene located in the vicinity of AGAP003327, (blue signal) in subdivision 17B. Arrows point at the hybridization signals. Arrowhead shows an additional minor signal from AGAP003327.
(TIF)

Figure S6 **Chromosome mapping of positive phage from the** ***An. merus*** **Lambda DASH II phage library.** **A**) FISH of Phage 6D to both proximal (13D) and distal (9A) 2R+^o^ breakpoints on the 2R arm of *An. gambiae* (red signals). **B**) Hybridization of Phage 6D to the proximal 2Ro breakpoint (9A/13D) in *An. merus*. **C**) FISH of Phage 6D to the unique locus 15B-16A on polytene chromosomes of outgroup species *An. stephensi*. **D**) Detailed mapping of Phage 6D to the proximal 2Ro breakpoint in the region 9A/13D and Phage 3B to the proximal 2Rp breakpoint in the region 9C on a highly polytenyzed chromosome 2R of *An. merus*. Arrowheads show an additional signal on 3L in *An. gambiae* (**A**) and *An. merus* (**B**).
(TIF)

Figure S7 **Unrooted trees of karyotype evolution in the** ***An. gambiae*** **complex recovered by the MGR program.** Each tree includes an outgroup species with different X chromosome arrangements: (**A**) X+, (**B**) Xbcd, and (**C**) Xag indicated with a blue font. The number of rearrangements that occurred on each edge is shown. The names of fixed inversions are shown in parentheses. A7–A11 are putative intermediate karyotypes. The second origin of 2Ro is highlighted with yellow in (**A**) and (**B**).
(TIF)

Table S1 **The BLASTN search of** ***An. merus*** **mate-paired sequencing reads detects the 2Ro and 2La inversion breakpoints in the** ***An. gambiae*** **AgamP3 assembly genome.**
(XLSX)

Table S2 **The output of Bowtie alignments using** ***An. merus*** **mate-paired sequencing reads confirms the positions of 2Ro inversion breakpoints in the** ***An. gambiae*** **genome.**
(XLSX)
