# Supplementary figures and images for: A New Chromosomal Phylogeny Supports the Repeated Origin of Vectorial Capacity in Malaria Mosquitoes of the Anopheles gambiae Complex
Source: PLoS Pathog. 2012 Oct 4;8(10):e1002960. doi: 10.1371/journal.ppat.1002960 (PMC3464210; doi:10.1371/journal.ppat.1002960)

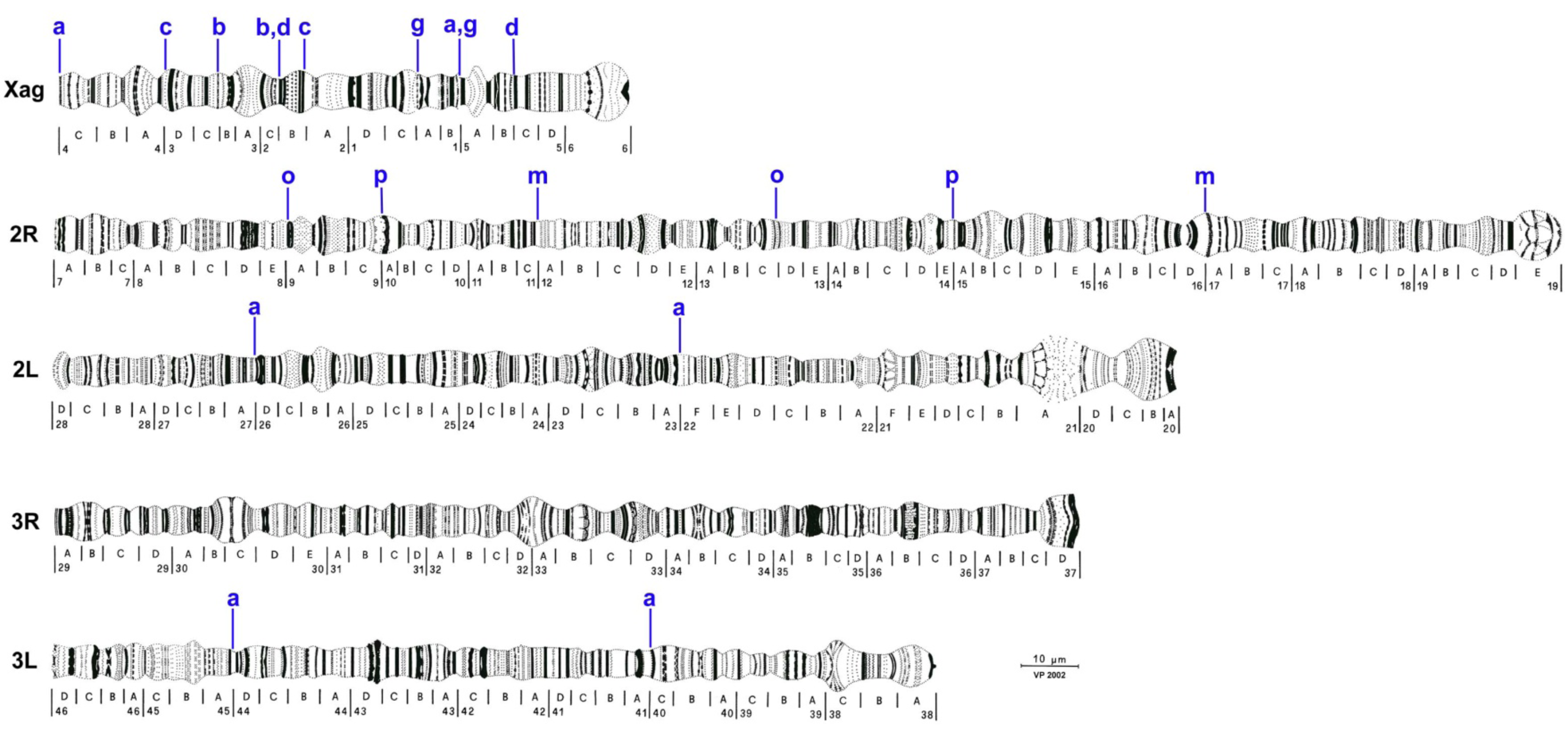

Supplement: Figure S1 — The 10 fixed paracentric inversions in sibling species of the An. gambiae complex. The positions of breakpoints are shown in blue with small letters above the chromosomes. (TIF) [file ppat.1002960.s001.tif]

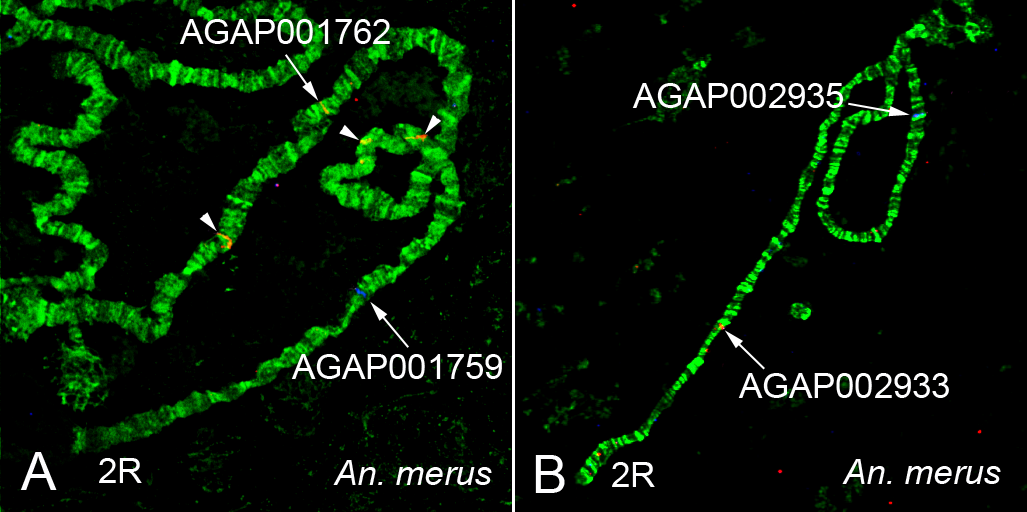

Supplement: Figure S2 — Physical mapping of genes at the 2Ro inversion breakpoints on polytene chromosomes of An. merus . A) FISH of AGAP001759 (blue signal) and AGAP001762 (red signal) to subdivisions 8E and 9A, which are located at the distal and proximal breakpoints, respectively. B) Localization of AGAP002933 (red signal) in the distal breakpoint (13C) and AGAP002935 (blue signal) in the proximal breakpoint (13D). Arrows point at the hybridization signals. Arrowheads show additional signals from AGAP001762. Chromosomes are counterstained with the fluorophore YOYO-1. (TIF) [file ppat.1002960.s002.tif]

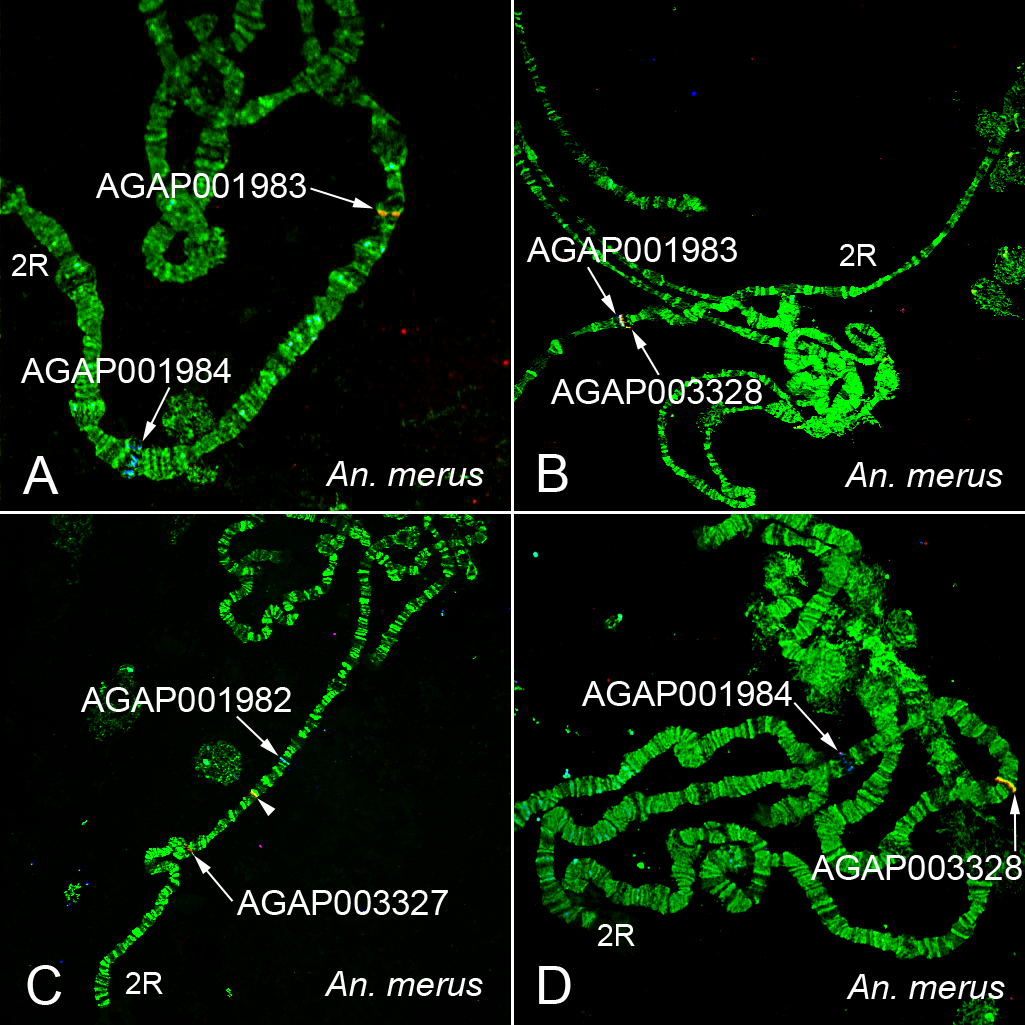

Supplement: Figure S3 — Physical mapping of genes at the 2Rp inversion breakpoints on polytene chromosomes of An. merus . A) FISH of AGAP001983 (red signal) and AGAP001984 (blue signal) to subdivisions 9C and 10A, which are located at the proximal and distal breakpoints, respectively. B) Localization of AGAP001983 (blue signal) and AGAP003328 (red signal) in the neighboring subdivisions 9C and 15A of the proximal breakpoint. C) FISH of AGAP003327 (red signal) with the distal breakpoint (10A) and of AGAP001982, the neighboring gene of AGAP001983, (blue signal) with the proximal breakpoint (9C). D) Mapping of AGAP001984 (blue signal) to the distal breakpoint (14E) and of AGAP003328 (red signal) to the proximal breakpoint (15A). Arrows point at the hybridization signals. Arrowhead shows an additional signal from AGAP003327. (TIF) [file ppat.1002960.s003.tif]

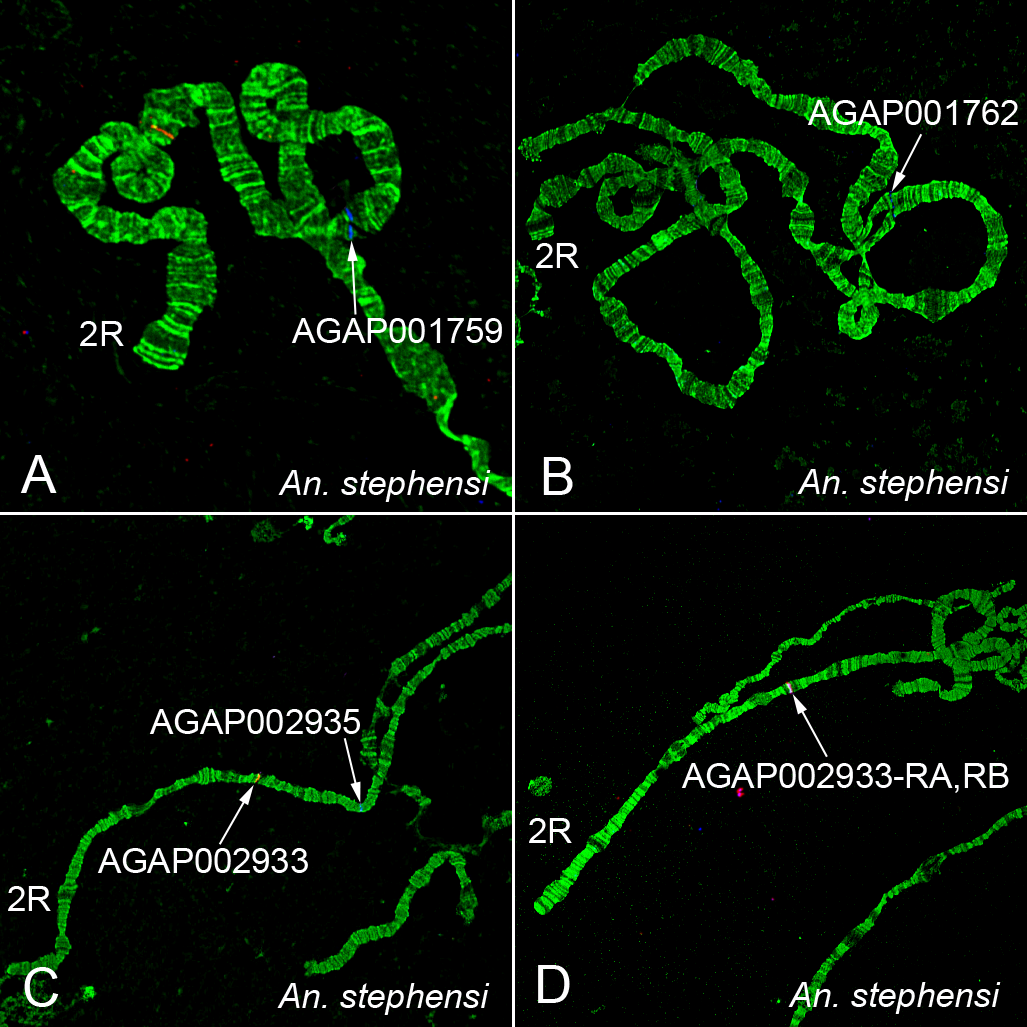

Supplement: Figure S4 — Physical mapping of genes from the 2Ro inversion breakpoints on polytene chromosomes of An. stephensi . A) FISH of AGAP001759 (blue signal) to subdivision 11AB. B) Localization of AGAP001762 (blue signal) in subdivision 15B-16A. C) FISH of AGAP002933 (red signal) with subdivision 11AB and of AGAP002935 (blue signal) in subdivision 15B-16A. D) Colocalization of probes derived from transcripts AGAP002933-RA (red signal) and AGAP002933-RB (blue signal) in subdivision 11AB. Arrows point at the hybridization signals. (TIF) [file ppat.1002960.s004.tif]

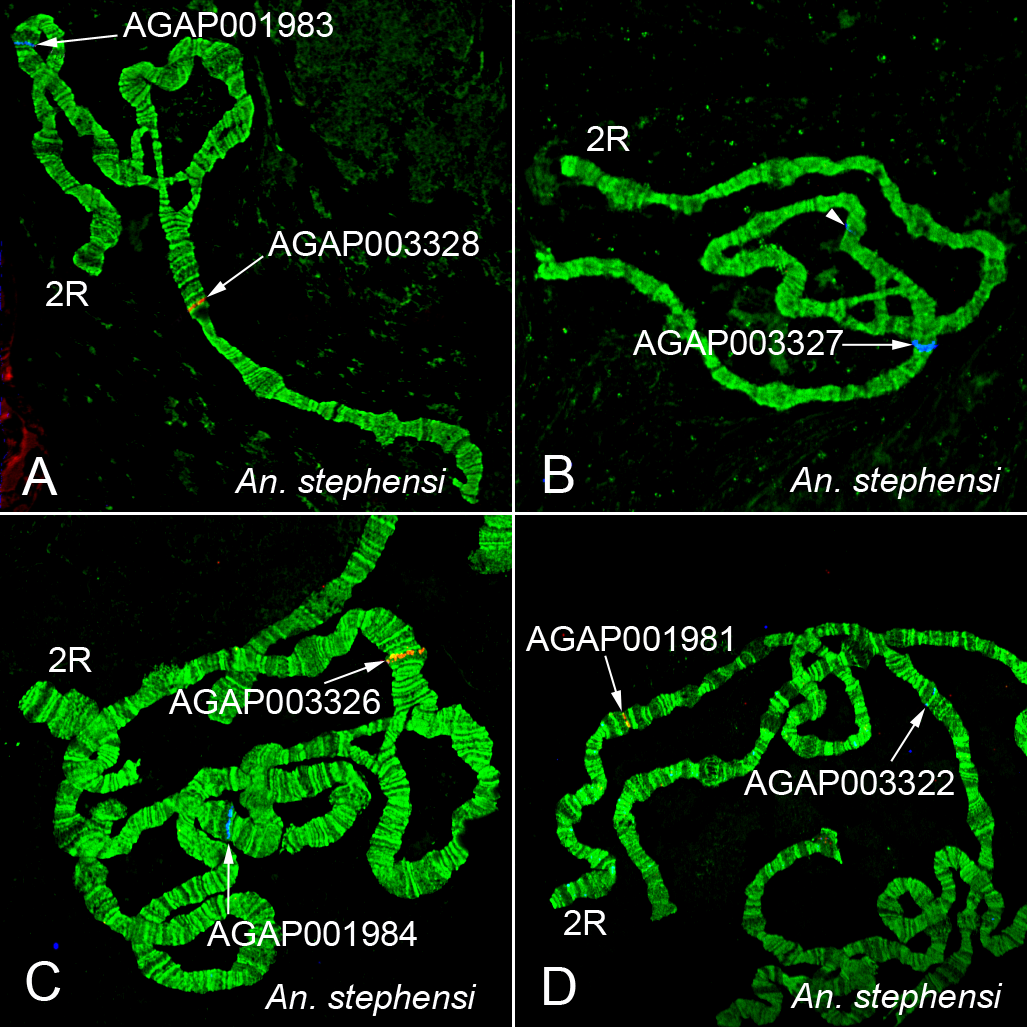

Supplement: Figure S5 — Physical mapping of genes from the 2Rp inversion breakpoints on polytene chromosomes of An. stephensi . A) FISH of AGAP001983 (blue signal) and AGAP003328 (red signal) to subdivisions 10A and 17C, respectively. B) Localization of AGAP003327 (blue signal) in subdivision 17B. C) FISH of AGAP001984 (blue signal) to subdivision 10A and of AGAP003326, the neighboring gene of AGAP003327, (red signal) to subdivision 17B. D) Mapping of AGAP001981, a gene located in the vicinity of AGAP001983, (red signal) in subdivision 10A and of AGAP003322, a gene located in the vicinity of AGAP003327, (blue signal) in subdivision 17B. Arrows point at the hybridization signals. Arrowhead shows an additional minor signal from AGAP003327. (TIF) [file ppat.1002960.s005.tif]

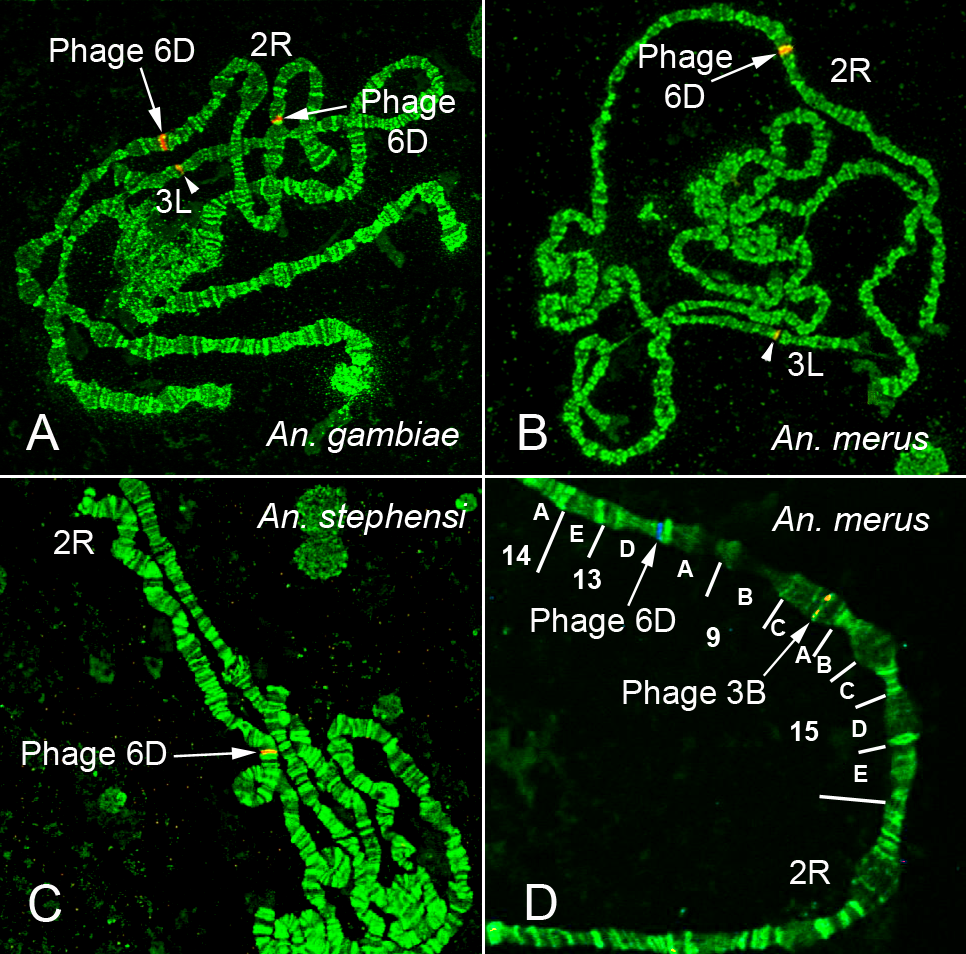

Supplement: Figure S6 — Chromosome mapping of positive phage from the An. merus Lambda DASH II phage library. A) FISH of Phage 6D to both proximal (13D) and distal (9A) 2R+o breakpoints on the 2R arm of An. gambiae (red signals). B) Hybridization of Phage 6D to the proximal 2Ro breakpoint (9A/13D) in An. merus. C) FISH of Phage 6D to the unique locus 15B-16A on polytene chromosomes of outgroup species An. stephensi. D) Detailed mapping of Phage 6D to the proximal 2Ro breakpoint in the region 9A/13D and Phage 3B to the proximal 2Rp breakpoint in the region 9C on a highly polytenyzed chromosome 2R of An. merus. Arrowheads show an additional signal on 3L in An. gambiae (A) and An. merus (B). (TIF) [file ppat.1002960.s006.tif]

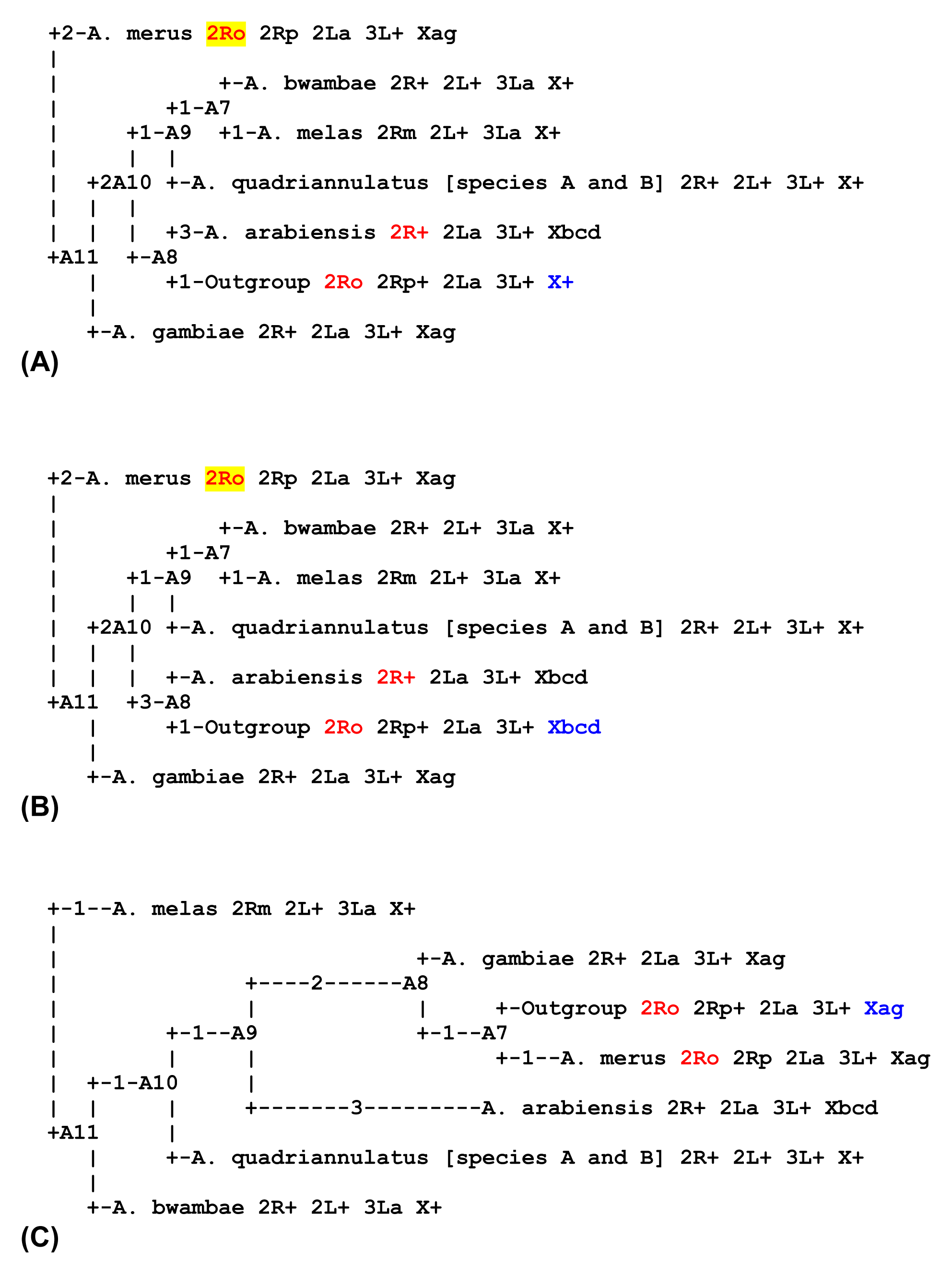

Supplement: Figure S7 — Unrooted trees of karyotype evolution in the An. gambiae complex recovered by the MGR program. Each tree includes an outgroup species with different X chromosome arrangements: (A) X+, (B) Xbcd, and (C) Xag indicated with a blue font. The number of rearrangements that occurred on each edge is shown. The names of fixed inversions are shown in parentheses. A7–A11 are putative intermediate karyotypes. The second origin of 2Ro is highlighted with yellow in (A) and (B). (TIF) [file ppat.1002960.s007.tif]
